# Cascade of diabetes care in Bangladesh, Bhutan and Nepal: identifying gaps in the screening, diagnosis, treatment and control continuum

**DOI:** 10.1038/s41598-023-37519-w

**Published:** 2023-06-24

**Authors:** Md Tauhidul Islam, Mieghan Bruce, Khurshid Alam

**Affiliations:** 1 Murdoch Business School, Murdoch University, Perth, WA 6150 Australia; 2 School of Veterinary Medicine and Centre for Biosecurity and One Health, Harry Butler Institute, Murdoch University, Perth, WA 6150 Australia

**Keywords:** Health policy, Health services

## Abstract

Diabetes has become a major cause of morbidity and mortality in South Asia. Using the data from the three STEPwise approach to Surveillance (STEPS) surveys conducted in Bangladesh, Bhutan, and Nepal during 2018–2019, this study tried to quantify the gaps in diabetes screening, awareness, treatment, and control in these three South Asian countries. Diabetes care cascade was constructed by decomposing the population with diabetes (diabetes prevalence) in each country into five mutually exclusive and exhaustive categories: (1) unscreened and undiagnosed, (2) screened but undiagnosed, (3) diagnosed but untreated, (4) treated but uncontrolled, (5) treated and controlled. In Bangladesh, Bhutan, and Nepal, among the participants with diabetes, 14.7%, 35.7%, and 4.9% of the participants were treated and controlled, suggesting that 85.3%, 64.3%, and 95.1% of the diabetic population had unmet need for care, respectively. Multivariable logistic regression models were used to explore factors associated with awareness of the diabetes diagnosis. Common influencing factors for awareness of the diabetes diagnosis for Bangladesh and Nepal were living in urban areas [Bangladesh-adjusted odd ratio (AOR):2.1; confidence interval (CI):1.2, 3.6, Nepal-AOR:6.2; CI:1.9, 19.9].

## Introduction

Increased blood glucose level is the leading risk factor for premature death, stroke, and heart disease worldwide, a major global health concern, regardless of income level^1^, although traditionally, it was seen as a problem common to high-income nations^2^. A projection up to 2030 on the global economic burden of diabetes revealed that the economic burden of diabetes would not decrease even if countries reach the Sustainable Development Goal (SDG) target for non-communicable diseases (NCDs) by 2030^3^. The analysis further estimated that the global financial burden of diabetes would increase from 1.3 trillion United States Dollar (USD) in 2015 to 2.2 trillion USD in 2030^3^.

In recent decades, diabetes has quickly become a significant public health challenge in South Asia. An estimation based on the data of the International Diabetes Federation (IDF) reported that in 2021, more than 90 million adults with diabetes lived in the South Asian region, increasing to over 150 million by 2045^4^. Unfortunately, despite the growing availability of evidence-based behavioural and pharmacotherapeutic interventions for treating and preventing diabetes, a substantial health service delivery gap in diabetes remains. Data from low and middle-income countries (LMIC) revealed that only 64.3% of those with diabetes had ever been tested with a blood glucose measurement, 44% were aware of their diagnosis, 38.4% received treatment, and only 22.8% had achieved disease control^5^. Therefore, the concept of the cascade of care has been proposed to examine the extent of health service delivery gaps as patients move from one stage to the next in the continuum of care. The cascade of care is a paradigm for assessing patient retention throughout various stages of care in order to have a positive treatment outcome ^6^. This method is most frequently utilised for the purpose of visualising the human immunodeficiency virus (HIV) care continuum in international contexts; however, it has also been applied to the visualisation of sexually transmitted infections, tuberculosis^6^, and, more recently, NCDs such as diabetes in the United States of America (USA), Samoa, Tanzania, and South Africa^7–10^. Overall, the cascade of care framework is a valuable tool for improving the quality of care for people with chronic conditions. Such as using this approach, the study from the USA found that three out of ten adults in the USA with diabetes remain undiagnosed, and later authors suggested developing education programs to improve the situation^8^. A recent study from Tanzania compared the drop off in each stage of cascade care for hypertension and diabetes and found less drop off in diabetes care compared to hypertension^10^.

In this paper, we used the cascade of care framework to summarise the evidence on diabetes screening, diagnosis, awareness, treatment, and control in three South Asian countries: Bangladesh, Bhutan, and Nepal. We also attempted to identify where losses occur in the continuum of care, and to investigate to what extent determinants influence rates of disease diagnosis in these three countries.

The fight against NCDs in the south-east Asia region has taken a significant leap forward by adopting the "Implementation Roadmap for Accelerating the Prevention and Control of NCDs in South-East Asia 2022–2030" during the Seventy-fourth session of the WHO Regional Committee. In this session, all member countries are targeted to provide access to affordable treatment for diabetes and blood glucose self-monitoring kits for 100% people with diabetes and take strategies to diagnose 80% of people with diabetes and improve the prevalence of glycaemic control to 80% among the people with diagnosed diabetes by 2030^11^. This contemporary study with the cascade care approach will support member countries, notably Bangladesh, Bhutan, and Nepal with baseline information to take necessary measures to achieve the target. Finally, a comparative perspective allows countries to explore what is achievable beyond national boundaries and simultaneously gain the necessary understanding before making any recommendations^12^. However, considering the data accessibility and availability of recent data, this study only employed the cascade care approach to Bangladesh, Bhutan, and Nepal.

## Method

### Data source

This study analysed data from three STEPwise approach to Surveillance (STEPS) surveys conducted in Bangladesh, Bhutan, and Nepal during 2018–2019. All three surveys were nationally representative, in which the multistage cluster sampling method was used to select a single individual from each sampled household. All three survey collected data from in-person interviews, physical examinations, and blood specimens. The STEPS survey applied the World Health Organization (WHO) NCD STEPS instrument, structured into STEP I, STEP II, and STEP III to measure the behavioural, anthropometric, and biological characteristics of the participants^13^.

### Bangladesh STEPS Survey, 2018

Bangladesh STEPS Survey, 2018, a cross-sectional study, was carried out from February to May 2018 among the adult population aged 18–69 years, including men and women residing in the households in all the divisions of Bangladesh. Sampling was multistage which used geographically stratified probability-based sampling. Considering the results of the Demographic Health Survey (DHS) and previous Bangladesh Bureau of Statistics (BBS) surveys, the person non-response rate, the household non-coverage rate, the design effect, security concerns, and the lack of clearance from local administration, the final adjusted sample size was 9900 adults from 495 primary sampling units (PSUs). At the end, 8185 respondents participated in the survey resulting in a response rate of 82.68%. A detailed description of the methodology of Bangladesh STEPS Survey, 2018 published elsewhere^14^.

### Bhutan STEPS Survey, 2019

In April 2019, Bhutan STEPS Survey 2019 was conducted. This was a cross-sectional study. Bhutan's target population of people ages 15 to 69 was represented by a sample size of 5632. Participants were chosen through a multistage cluster sampling that used a mix of probability proportionate to size (PPS) and systematic random sampling. The sampling frame was taken from the 2017 Population and Housing Census of Bhutan. In rural areas, the primary sampling unit (PSU) was a "Gewog", or county, and in urban areas, it was a "thromde", or town. A total of 88 PSUs were chosen, 55 from rural areas and 33 from cities. Using the PPS method, four Secondary Sampling Units (SSUs) were chosen from each PSU. This made a total of 352 SSUs (220 from rural and 132 from urban). At the end, a total of 5, 575 people were interviewed resulting a response rate of 99%. Further information on the methodology of Bhutan STEPS Survey, 2019 is available elsewhere^15^.

### Nepal STEPS Survey, 2019

Nepal STEPS Survey 2019 was a cross-sectional survey. Adults between the ages of 15 and 69 who were eligible for the survey were interviewed between February and May 2019. While selecting the respondents for the survey, the current federal structure of Nepal was taken into account so that the results could be used at the provincial level. Multistage cluster sampling was used to choose 6475 eligible participants from each of Nepal's seven provinces. At the first stage, 37 PSUs from each province were selected from the 259 wards. Using systematic random sampling, 25 households per PSU were chosen from the list of households. At last, 5593 respondents participated in the survey resulting a response rate of 86.38%. Further information on the sampling process is available elsewhere^16^.

### Blood glucose measurement

All three surveys collected blood samples from fasting participants. In Bangladesh 5 ml of blood was collected by disposable syringe from antecubital vein. Then 2 ml of this blood was transferred to Fluoride-oxalate vacutainer for serum glucose testing. All the blood samples in Bangladesh were tested in the laboratory using biochemistry auto analyzer (Selectrao Pro M). Unlike Bangladesh, both Bhutan and Nepal used CardioChek pa analyser and capillary whole blood to measure blood glucose on the spot^14–16^.

### Physical measurement: anthropometry

In Bangladesh and Bhutan portable stadiometers were used to measure the height following standard protocol. Nepal used portable standard stature tape to measure the height. For the height measurement in all countries, respondents were asked to remove footwear (shoes, slippers, sandals) and any hat or hair ties. Respondents were requested to stand on the stadiometer facing the interviewer with their feet together and knees straight. They were asked to look straight ahead and not tilt their head up, making sure that their eyes were at the same level as their ears. Height was recorded in centimetres. In all three countries weight was measured with portable digital weighing scale. The instrument was placed on a firm, flat surface. Participants were requested to remove their footwear and socks, wear light clothes, stand on the scale with one foot on each side of the scale, face forward, place arms idly at their side and wait until asked to step off. Weight was recorded in kilograms^14–16^.

### Sample construction

Analysis reported here was restricted to diabetic patients only since this study only looked at the cascade of diabetes care in these three countries. Participants were considered diabetic if participants with a fasting blood sugar ≥ 126 mg/dl or those currently taking medications to lower blood sugar ^17^. Participants with missing data on blood glucose readings were excluded from the analysis, even though they have the information about taking anti-diabetic medication. Finally, 677, 181, and 343 participants were included in the analysis from Bangladesh, Bhutan, and Nepal, respectively.

### Outcome variables

**Figure 1:**
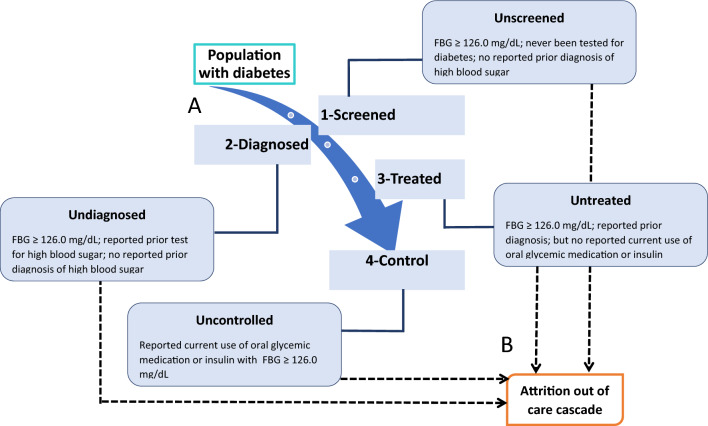
The diabetes cascade of care. Blue downward arrow (**A**) denotes stepwise progression in the diabetes care cascade. Dashed arrow (**B**) demonstrates stepwise attrition along the diabetes care cascade.

The outcome variables were diabetes screening, awareness, treatment, and control, which are collectively defined as the cascade of diabetes care (Fig. 1). To assess hypertension screening, the surveys asked if the individual participants had ever had their blood glucose measured by a doctor or another health worker. Participants were considered diagnosed if they knew they had high blood sugar, which had to have been diagnosed by a doctor or another health worker. Diabetes treatment was defined as the use of any oral hypoglycaemic agents or insulin to lower blood glucose in the last two weeks. Diabetes was controlled, if the participants had a blood sugar below 126 mg/dL^17^.

### Explanatory variables

This study will only observe the determinants that influence disease diagnosis rates in three South Asian countries. Considering that explanatory variables were chosen based on the extensive literature review and availability of variables in the datasets. Explanatory variables were age, sex, place of residence, years of education, occupation, visit to a health facility in the last 12 months, awareness of hypertensive status, and body mass index (BMI). Age was categorised into two categories (< 40 years and ≥ 40 years). Years of education were classified as 0–5 years of education, 6–12 years of education, and 13 years of education or above. Participants were considered aware of their hypertension if they knew they had high blood pressure, which had to have been diagnosed by a doctor or another health worker. Participants were asked whether they have visited a healthcare facility in the last 12 months, and the response options were ‘Yes’ and ‘No’. Participant’s BMI was categorised using an Asian cut-off: (0 = normal (BMI between 18.5 and < 23.0 kg/m^2^); 1 = underweight (BMI < 18.5 kg/m^2^; 2 = overweight BMI between 23.0 and < 27.5 kg/m^2^; 3 = obesity BMI ≥ 27.5 kg/m^2^)^18^.

### Statistical analysis

This study summarised participants’ sociodemographic and clinical characteristics using descriptive statistics, reporting the medians and interquartile ranges for continuous variables (non-normal distribution) and proportions and accompanying 95% confidence intervals (95%CIs) for categorical variables. Following that care cascade was developed to investigate the unmet need for diabetes care and find gaps to intervene across the domains of screening, diagnosis, treatment and control. Care cascades were constructed for five progressive stages: (1) total diabetes among the study population; (2) among those classified as having type 2 diabetes, screened for diabetes through blood glucose measurements; (3) among those who screened their condition, the proportion aware about the diagnosis of diabetes, confirmed by a doctor or health professionals; (4) among those aware of their condition, the proportion currently on medication for that condition (last 2 weeks); and (5) among those on medication, the proportion who achieved recommended blood sugar level. Then, these five categories were used to determine the percentage of individuals with diabetes lost across the care cascade according to the methods outlined by Stokes et al.^7^. The proportion of participants who reached each stage was calculated using the number of participants from the subsequent stage as the numerator and the total number of diabetic cases as the denominator in each case. Participants “lost” were calculated by subtracting the proportion of participants who reached each subsequent stage from the total number of participants who reached the previous stage. Unmet need was calculated by subtracting the proportion of participants with controlled diabetes from the total number of diabetic patients. Among participants with diabetes, sociodemographic correlates of diabetes diagnosis were examined using bivariate and multivariable logistic regression models. Bivariate measures were presented as unadjusted odds ratio (OR), and measures from multivariable logistic regression were presented as adjusted OR (AOR). However, it is recommended to have multilevel or mixed-effect logistic regression because of the clustered sample survey data^19^. Considering the containment of the analysis to the participants with diabetes, lack of representation (1–3 participants) from each PSU, and low (close to zero) intracluster correlation coefficients (ICC) at the stratum level (Supplemental Table 1) of each of the countries for the awareness of diabetes diagnosis, indicating that there were very minimum variations in outcome between the clusters^20^. Therefore, this study decided to perform the multivariable logistic regression. Statistical analyses were performed with STATA version 17.0 (basic edition). P-values (two-sided) < 0.05 were considered statistically significant. Error represents 95% confidence interval (CI). Sample weights were incorporated in all analyses to adjust for unequal probabilities of selection and non-response. The results were graphically represented using ggplot2 package of R.

## Result

### Sociodemographic and clinical characteristics

Table 1 presents sociodemographic and clinical characteristics by country. Among all three countries, Bhutan had higher proportion of participants (76.2%) who were aged 40 years and above compared to Bangladesh (64.6%) and Nepal (58.1%). In all countries, the proportion of males in the sample was higher than females. The proportion of participants included with no formal education to 5 years of education ranged from 53.4% in Bangladesh to almost 61.6% among the participants in Bhutan. In Bangladesh and Nepal, respondents were mostly from rural area (Bangladesh-68.5% and Nepal-83.6%). On the contrary in Bhutan (51.9%), majority of the respondents were from urban areas. Nepali participants had the largest median for fasting blood glucose (FBG) (145; IQR:130–172), followed by Bangladesh (144; IQR:131–192), and Bhutan (130; IQR:103–165). Nearly three out of four participants in Bhutan were obese, and in Bangladesh, one out of two participants were obese. Compared to Bangladesh and Bhutan, Nepal had the lowest proportion of obesity (two out of five) among the participants.

**Table 1:** Sociodemographic and clinical description (weighted) of the final analytic study sample (N = 1201).

Characteristics	Bangladesh (N = 677)	Bhutan (N = 181)	Nepal (N = 343)
	Percentage (95%CI)	Percentage (95%CI)	Percentage (95%CI)
Age (years)			
Median (IQR)	47.0 (35.0–60.0)	50.0 (41.0–60.0)	43.0 (32.0–53.0)
< 40 years	35.4 (29.3, 41.9)	23.8 (14.9, 35.7)	41.9 (34.2, 50.0)
≥ 40 years	64.6 (58.0, 70.7)	76.2 (64.2, 85.0)	58.1 (49.9, 65.7)
Sex			
Male	50.1 (43.4, 56.8)	52.0 (42.1, 61.6)	50.5 (42.8, 58.0)
Female	49.9 (43.2, 56.6)	48.0 (38.3, 57.8)	49.5 (41.9, 57.1)
Place of residence			
Urban	31.5 (26.4, 37.1)	51.9 (42.0, 61.6)	16.4 (11.9, 22.0)
Rural	68.5 (62.9, 73.5)	48.1 (38.4, 57.9)	83.6 (77.9, 88.0)
Years of education^a^			
0–5	53.4 (46.5, 60.0)	61.6 (51.4, 70.7)	56.1 (48.1, 63.7)
6–12	34.8 (28.9, 41.2)	27.2 (19.3, 36.8)	37.8 (30.4, 45.6)
13 and above	11.8 (8.0, 17.0)	11.2 (6.3, 19.2)	6.1 (2.8, 12.8)
Occupation^a^			
White collar	15.5 (11.2, 20.9)	24.9 (17.2, 34.4)	11.8 (7.6, 17.6)
Blue collar	28.6 (23.2, 34.6)	43.0 (33.2, 53.4)	23.9 (18.1, 30.7)
Pink collar	45.1 (38.4, 51.9)	23.5 (17.0, 31.4)	50.9 (43.3, 58.5)
Unemployed	10.8 (7.4, 15.4)	8.6 (4.9, 14.4)	13.4 (8.8, 19.8)
FBG (mg/dl)			
Median (IQR)	144 (131–192)	130 (103, 165)	145 (130, 172)
Aware of hypertensive status			
Yes	40.9 (33.7, 48.5)	47.4 (37.5, 57.5)	31.6 (24.1, 40.2)
BMI			
Normal/underweight	32.4 (26.4, 38.9)	14.3 (9.0, 21.8)	39.4 (32.1, 47.0)
Overweight	17.9 (13.8, 22.9)	11.3 (6.9, 17.7)	27.8 (21.3, 35.3)
Obese	49.7 (42.9, 56.4)	74.4 (65.7, 81.5)	32.8 (26.1, 40.3)

^a^Missing, years of education; Bangladesh = 25, Bhutan = 4, Nepal = 6, occupation; Nepal = 1, BMI; Bangladesh = 7, Bhutan = 1, Nepal = 2.

### Cascade of care: screening, diagnosis, treatment, and control of diabetes

**Figure 2:**
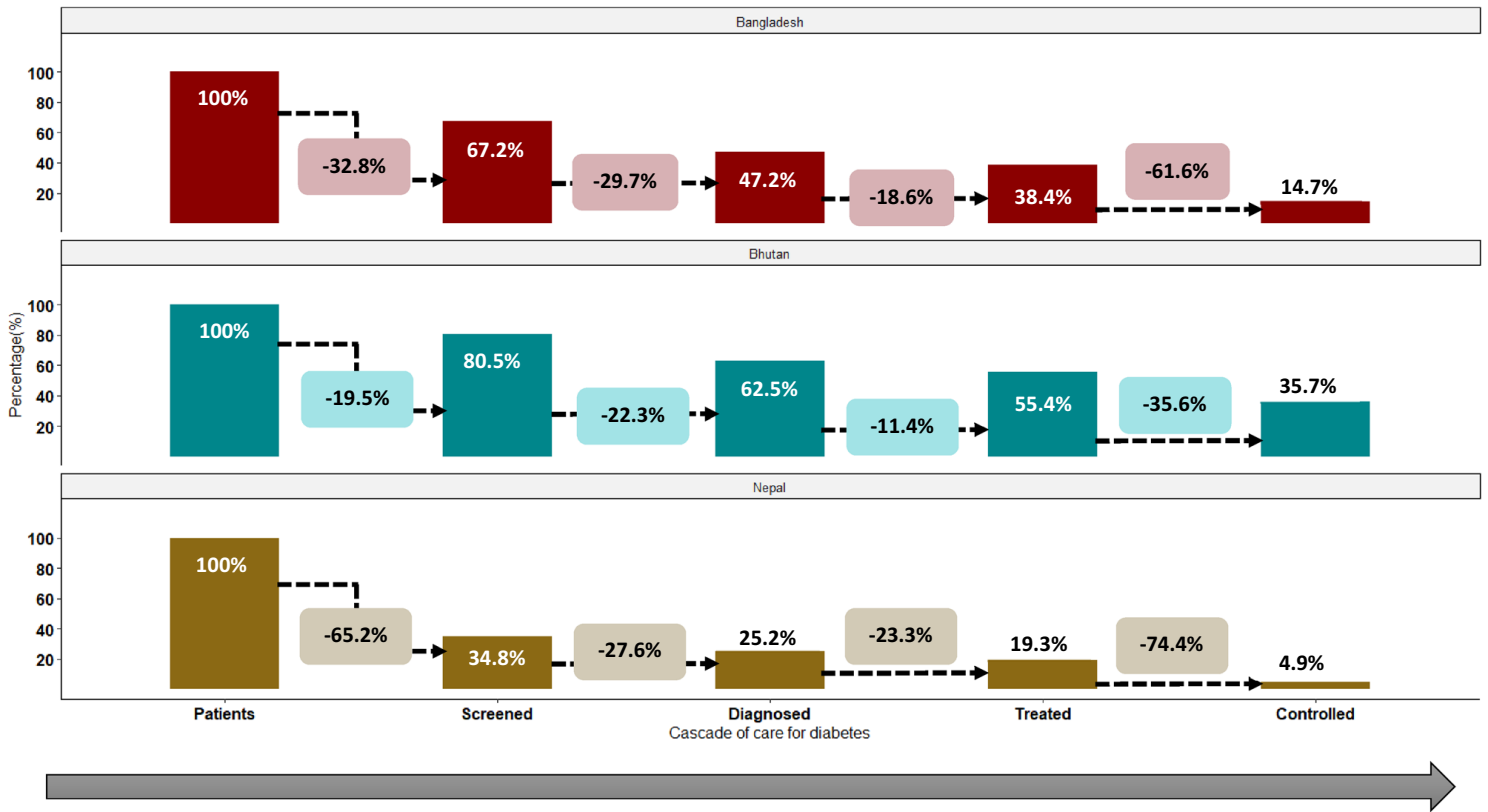
The diabetes care cascade in Bangladesh, Bhutan, Nepal.

Figure 2 demonstrates the change between steps of the diabetes care cascade. The first stage of this care cascade was investigated through the question, “Have you ever had your blood sugar (Diabetes) measured by a doctor or other health worker?”. Among those with diabetes in Bhutan, 80.5% reported that they had ever been screened, and this is highest while comparing to Bangladesh (67.2%) and Nepal (34.8%). The attrition observed for Bhutan, Bangladesh, and Nepal were 19.5%, 32.8% and 65.2%, respectively. Among those who self-reported ever being screened in Bangladesh, 70.3% were aware of their diagnosis (told that they were informed by the doctors or health professionals that they have diabetes). It was 77.7% and 72.4% in Bhutan and Nepal, respectively. From screening to diagnosis, the highest loss was observed for Bangladesh (29.7%), followed by Nepal (27.6%) and Bhutan (22.3%). Among those who self-reported a prior diagnosis in Bhutan, 88.6% were currently on medication (last 2 weeks) to treat or control their diabetes, whereas in Bangladesh 81.4% and Nepal 76.7% people were currently taking oral hypoglycaemic agents or insulin to control their diabetes. The highest transition from awareness of diagnosis to treatment was observed for Nepal (23.3%), followed by 18.6% for Bangladesh and 11.4% for Bhutan. Further, among those who received treatment for diabetes in Bangladesh and Nepal, 38.4% and 25.6% controlled their blood sugar for diabetes, respectively. Control rate was the highest in Bhutan (35.7%) in comparison to Bangladesh (14.7%) and Nepal (4.9%). In the last stage an 74.4% loss was observed for Nepal, and slightly lower loss were observed for Bangladesh (61.6%). In Nepal, 95.1% of all study participants had an unmet need for diabetes care, followed by 85.3% in Bangladesh and 64.3% in Bhutan.

### Factors influencing awareness of diabetes diagnosis

**Figure 3:**
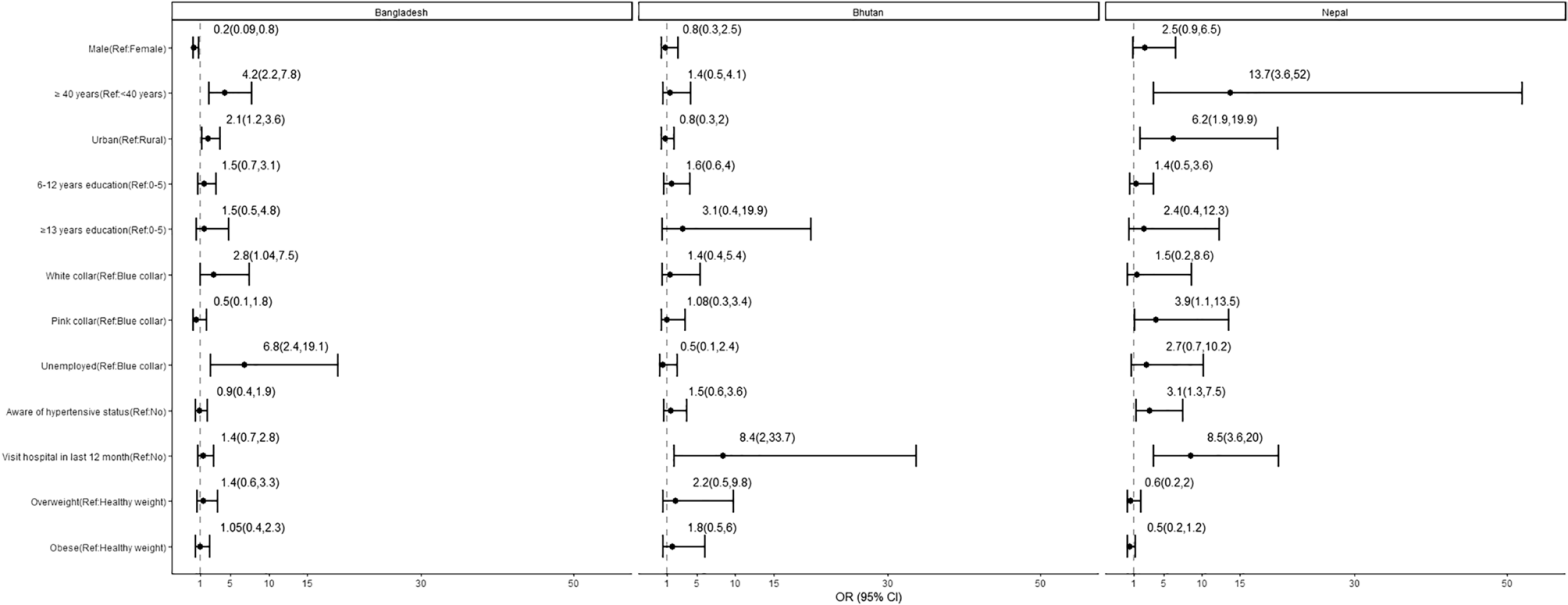
Multivariable association between independent factors and awareness of diabetes diagnosis. Awareness was based on participant report of a prior diabetes diagnosis, and OR adjusted for all other variables presented (sex, age category, place of residence, years of education, occupation, awareness of hypertensive status, hospital visit in last 12 months, and BMI category).

Figure 3 illuminates result from adjusted multivariable logistic regression that in Bangladesh, those who were male were 80% less likely (AOR: 0.20; 95%CI:0.09, 0.8) to aware of their diabetic condition. However, for Nepal and Bhutan this association did not reach statistical significance (p < 0.05). Being aged 40 years and above was associated with more than four times higher odds in Bangladesh (AOR: 4.2; 95% CI: 2.2, 7.8) and nearly 14 times higher odds in Nepal (AOR: 13.7; 95% CI: 3.6, 52.0) of being aware of the diabetic condition. Being a residence of urban area compared to rural area possessed twice higher odds of being aware of the diabetic condition in Bangladesh (AOR: 2.1; 95% CI: 1.2, 3.6), and for Nepal, it was more than six times higher odds (AOR: 6.2; 95% CI: 1.9, 19.9). In Bangladesh workers with white collar job category had nearly three times (AOR: 2.8; 95% CI: 1.04, 7.5) higher odds to be aware of their diabetic condition compared to blue collar professionals, whereas in Nepal, pink collars had about four times higher odds (AOR: 3.9; 95% CI: 1.1, 13.5) to be aware of their diabetic condition. In Nepal those who were aware about their hypertensive condition had three times higher odds (AOR: 3.1; 95% CI: 1.3, 7.5), and those who visited the hospital in the last 12 months had more than eight times higher odds (AOR: 8.5; 95% CI: 3.6, 20.0) of being aware about their diabetic status. Similarly in Bhutan those who went to hospital in 12 months had more than eight times higher odds (AOR: 8.4; 95% CI: 2.0, 33.7) of being aware of the diagnosis of diabetes mellitus. The unadjusted model for each country is presented in Supplemental Table 2.

## Discussion

To the best of found knowledge, this research is the first analysis of health system performance in diabetes management in South Asia, particularly for Bangladesh, Bhutan and Nepal applying the concept of cascade care (from screening to control of diabetes). However, a secondary analysis with data from 28 countries (timeframe: 2008–2016) included Bangladesh, Bhutan and Nepal, but the study portrayed the result based on geographic region and national income, not individual country-specific^5^. Overall, we found that the health system's performance of Bangladesh and Nepal for managing diabetes is characterised by large care losses at the stage of diabetes screening, and control, and moderate rates of losses during diagnosis and treatment. Compared to Bangladesh and Nepal, Bhutan’s performance is better, characterised by large care losses at diagnosis and control stage and moderate losses during screening and treatment. Total unmet need for diabetes care (defined as the sum of those not tested, tested but undiagnosed, diagnosed but untreated, and treated but with diabetes not controlled) was high in all these three countries.

A recent and similar investigation in a LMIC country, Samoa, reported around 99% unmet need for diabetes care. Unlike these three South Asian countries, Samoa showed large care losses at the stage of diagnosis of diabetes (79.6%), but the similarity was observed regarding losses at the stage of control (95.2%)^9^. An upper-middle income country, South Africa demonstrated a large loss at the stage of screening and control of diabetes. Regarding loss at the stage of screening of diabetes, Bangladesh (32.8%) and Bhutan (19.5%) performed better than South Africa (49%). On the other hand, loss at the stage of control in Bangladesh (61.6%) and Nepal (74.4%) is higher in comparison to the loss of South Africa (49%). A study based on 28 countries between 2008 and 2016 demonstrated that in LMICs, major losses happened at the stage of screening and control of diabetes^5^. The study further observed the cascade of care regionally, and they found that Asian countries (including Bangladesh, Bhutan and Nepal) also experienced large care losses at the stage of screening and control of diabetes, whereas Middle East Asian countries experienced major losses at the stage treatment^5^. These losses at the stage of control could be the result of factors on the demand side, such as lack of patient awareness and engagement, inability to afford care, or sociocultural barriers^21^; or they could be the result of factors on the supply side, such as lack of services, lack of responsiveness in the services that are provided, or geographic inaccessibility^22^. A recent investigation based on 55 LMICs reported that less than one in ten patients with diabetes receive comprehensive diabetes treatment (both pharmacological and non-pharmacological) based on guideline^23^. Contributing factors to this effective treatment gap also include limited health literacy and anti-diabetic medication non-adherence^24,25^. Better performance of Bangladesh and Bhutan in regard to screening could be explained by the vertical program designed by each country. The Government of Bangladesh established NCD corner at primary care level, and a non-government organization (NGO) named Diabetic Association of Bangladesh (BADAS) provide diabetes care with centres in every district^26^. Bhutan is also implementing National Diabetes Control Programme since 1996^27^. On contrary, Nepal neither have any nationwide robust vertical programme nor national guideline for the prevention and treatment of diabetes^28,29^.

It is essential to highlight that all three countries also experienced substantial care losses at treatment stage compared to South Africa’s experience (only 6% loss)^7^. These losses at this stage could be linked with the high out-of-pocket (OOP) expenditure (56 USD in 2019) for health in South Asia, whereas in South Africa, OOP was 5.6 USD in 2019^30^. Further, an investigation in Bangladesh identified socioeconomic disparities in receiving anti-diabetic medication^31^. A study on Nepal’s health system capacity identified low availability, poor affordability and accessibility of essential medicines and diagnostics in regard to basic health services to cardiovascular disease and diabetic patients^32^. Compared to Bangladesh and Nepal, Bhutan is performing better in managing NCDs; however, all essential anti-diabetic drug is also not available at primary healthcare level of Bhutan, which might contribute to losses at treatment stage^33^. Further it is important to note that Universal Health Coverage (UHC) index (capture the service coverage and financial protection dimensions) score 2019 for NCDs in Bangladesh, Bhutan, and Nepal is 56, 47 and 58, respectively, whereas Canada and Australia scored 80 and 73, respectively^34^.

In both Bangladesh and Nepal, those people who were living in urban areas were more likely aware about their diabetic condition which could be explained by higher health service readiness for diabetes in urban area compared to rural area in both countries^35,36^. It is also argued that people in urban areas have more pronounced healthcare values than people in rural areas^37^. Study findings of Bangladesh are consistent with the recent findings drawn from Bangladesh Demographic Health Survey (BDHS)^38^. Similar work was not found for Nepal, but a nationwide study on hypertension found that urban participants were more likely to aware about their hypertensive status compared to rural participants^39^. Again, in Bangladesh and Nepal, those who were 40 years and above were more likely to aware about their diabetic condition which might be the result of following the implementation tool Package of Essential Non-communicable (PEN) diseases interventions for primary health care in low-resource settings by Bangladesh and Nepal^40,41^, where age threshold for screening diabetes was 40 years^42^. However, recently USA suggested lowering the threshold to 35 for diabetes^43^, and India suggested that screening for diabetes should be initiated from 25 years^44^. In Bangladesh, people with white collar jobs were more likely to aware about their diagnosis compared to their blue collar counterparts which indicated the socio-economic disparity that exists in the management of diabetes in Bangladesh^31^. Attending physicians were a major source of awareness for diabetic patients, reflected in the result of Bhutan and Nepal, and consistent with similar studies in this arena^45,46^.

By examining the cascade of care, this study identified potential gaps in screening, diagnosis, treatment, and control rates for diabetes. This can inform researchers and policymakers to implement targeted screening programs and diagnostic initiatives, leading to earlier detection and improved management of the condition. Understanding how diabetes care works in different countries gives a more complete picture of the problems the world is facing. It can help to come up with global strategies, policies, and rules for better managing and controlling of diabetes. Thus, this study’s result could have significant contribution to global health system’s response to diabetes care.

The primary strength of this study that it used nationally representative data with comparatively larger sample sizes. STEPS surveys follow the standard framework and methods of the WHO STEPwise Approach to Noncommunicable Disease Risk-Factor Surveillance to collect nationally representative data from the study countries. A further advantage is the utilisation of a care cascade to provide population-level estimates of the diabetic patients who make it through each stage of the care continuum, starting with screening and ending with control.

However, there are a few limitations to note. In this study, cascade care for Bangladesh, Bhutan and Nepal was developed using one method, but there are a few different ways that cascades of care can be constructed, and the method that this study chose to use was a fixed denominator rather than a conditional or time-series analysis. Furthermore, certain elements of the survey questionnaire limited our examination of the ‘screening’, ‘diagnosis and ‘treatment’ stage in the care cascade because of its ‘self-reported’ nature, making it vulnerable to recall bias. It would be better if the response of the participants could be cross checked with hospital record. It was also argued that the cascade of care is a quantitative method for tracking the progression of persons with a given ailment through the health care system, but it does not provide full insight into the deeper understanding of events that may contribute to the loss of treatment. Therefore, further country specific mixed-method studies are recommended to identify the contextual factors that might contribute to the loss in the diabetes care cascade of Bangladesh Bhutan and Nepal. Besides that, only one glucose test is used to decide if someone has diabetes which could have made the inaccurate identification of patients with diabetes. Specifically, the lack of an oral glucose tolerance test (OGTT) could have missed potential participants with diabetes. Moreover, unavailability of HbA1C measurement put this study in a challenging position to identify the participants with controlled blood sugar, and might end up with overestimating rate of control^47^.

It was planned to include a separate multivariable logistic regression to observe the factors associated with the unmet need for diabetes care. However, the proportion of those who received the necessary diabetic care was insufficient for this analysis. Further, it would be better if this study observed the diabetes cascade of care by different age groups in each country since age is a major determinant of diabetes, and it differs across the countries^48^. However, this sub-group analysis was avoided due to insufficient samples in each age group.

## Conclusion

In conclusion, this study witnessed high level of unmet need for diabetes care among the adults of three South Asian countries, Bangladesh, Bhutan, and Nepal with the biggest window in control of diabetes. This analysis offers policy relevant insights to improve the health system’s response to diabetes care. To enhance the efficiency of diabetes care and the quality of patient outcomes, future experimental studies could be conducted to boost performance at each stage of the care cascade.

## Supplementary Information

Supplementary Table 1.

Supplementary Table 2.

## Data Availability

Data are available for public at the website of World Health Organization’s NCD Microdata Repository(https://extranet.who.int/ncdsmicrodata/index.php/home#:~:text=The%20WHO%20NCD%20microdata%20repository, for%20NCD%20prevention%20and%20control). Following instruction, data are available to download.
