# Supplementary material for: Cascade of diabetes care in Bangladesh, Bhutan and Nepal: identifying gaps in the screening, diagnosis, treatment and control continuum
Source: Sci Rep. 2023 Jun 24;13:10285. doi: 10.1038/s41598-023-37519-w (PMC10290703; doi:10.1038/s41598-023-37519-w)
Supplement: Supplementary file 1 — Supplementary Table 1. [file 41598_2023_37519_MOESM1_ESM.docx]

Supplemental Table 2: Univariate association between study variables and awareness of diabetes diagnosis

| **Influencing factors** | **Bangladesh** | **Bhutan** | **Nepal** |
| --- | --- | --- | --- |
|  | **OR (95%CI)** | **OR (95%CI)** | **OR (95%CI)** |
| **Age** | | | |
| < 40 years | 1 [Reference] | | |
| ≥ 40 years | 4.9 (2.7,8.8)* | 2.5 (0.8,7.9) | 9.2 (3.5,24.2)* |
| **Sex** | | | |
| Female | 1 [Reference] | | |
| Male | 0.7 (0.4,1.2) | 0.9 (0.4,2.3) | 1.4 (0.7,2.8) |
| **Place of residence** | | | |
| Rural | 1 [Reference] | | |
| Urban | 2.1 (1.3,3.5)* | 0.9 (0.3,2.1) | 2.5 (1.1,5.5)* |
| **Years of education^α^** | | | |
| 0-5 | 1 [Reference] | | |
| 6-12 | 1.2 (0.7,2.2) | 1.6 (0.6,4.1) | 0.9 (0.4,2.0) |
| 13 and above | 1.4 (0.5,3.6) | 2.8 (0.6,11.8) | 1.01 (0.2,4.5) |
| **Occupation^α^** | | | |
| White collar | 1 [Reference] | | |
| Blue collar | 2.6 (1.1,5.8)* | 3.2 (1.1,9.5) * | 1.04 (0.2,3.8) |
| Pink collar | 2.1 (1.1,3.9)* | 2.0 (0.7,5.8) | 1.3 (0.6,3.1) |
| Unemployed | 4.7 (1.9,11.6)* | 0.8 (0.2,3.1) | 1.1 (0.3,3,5) |
| **Aware of hypertensive status** | | | |
| No | 1 [Reference] | | |
| Yes | 1.8 (0.9,3.5) | 2.1 (0.8,5.0) | 2.8 (1.2,6.1)* |
| **Visit hospital in last 12 month** | | | |
| No | 1 [Reference] | | |
| Yes | 2.1 (1.1,3.8)* | 12.9 (3.0,54.2)* | 10.9 (5.0,23.6)* |
| **BMI** | | | |
| Normal/Underweight | 1 [Reference] | | |
| Overweight | 1.2 (0.6,2.5) | 2.8 (0.6,11.7) | 0.6 (0.2,1.6) |
| Obese | 1.4 (0.7,2.7) | 2.3 (0.7,7.0) | 1.02 (0.4,2.1) |
| *P value less than 0.05 | | | |
