# Supplementary material for: Cascade of diabetes care in Bangladesh, Bhutan and Nepal: identifying gaps in the screening, diagnosis, treatment and control continuum
Source: Sci Rep. 2023 Jun 24;13:10285. doi: 10.1038/s41598-023-37519-w (PMC10290703; doi:10.1038/s41598-023-37519-w)
Supplement: Supplementary file 2 — Supplementary Table 2. [file 41598_2023_37519_MOESM2_ESM.docx]

Supplemental Table 1: Intracluster correlation coefficient (ICC) for the awareness of diabetes diagnosis at the level of the stratum

| **Country** | **Level** | **ICC** | **Standard error** |
| --- | --- | --- | --- |
| Bangladesh | Stratum | 0.02 | 0.02 |
| Bhutan | Stratum | 6.69e-34 | 1.07e-17 |
| Nepal | Stratum | 1.40e-30 | 1.40e-30 |
